# Obsessive compulsive disorder in very young children – a case series from a specialized outpatient clinic

**DOI:** 10.1186/s12888-020-02780-0

**Published:** 2020-07-11

**Authors:** Veronika Brezinka, Veronika Mailänder, Susanne Walitza

**Affiliations:** Department of Child and Adolescent Psychiatry and Psychotherapy, University Hospital of Psychiatry Zurich, University of Zurich, Neumünsterallee 3, 8032 Zurich, Switzerland

**Keywords:** Obsessive compulsive disorder, Early childhood, Case report, CBT, Family accommodation

## Abstract

**Background:**

Paediatric obsessive-compulsive disorder (OCD) is a chronic condition often associated with severe disruptions of family functioning, impairment of peer relationships and academic performance. Mean age of onset of juvenile OCD is 10.3 years; however, reports on young children with OCD show that the disorder can manifest itself at an earlier age. Both an earlier age of onset and a longer duration of illness have been associated with increased persistence of OCD. There seems to be difficulty for health professionals to recognize and diagnose OCD in young children appropriately, which in turn may prolong the interval between help seeking and receiving an adequate diagnosis and treatment. The objective of this study is to enhance knowledge about the clinical presentation, diagnosis and possible treatment of OCD in very young children.

**Case presentation:**

We describe a prospective 6 month follow-up of five cases of OCD in very young children (between 4 and 5 years old). At the moment of first presentation, all children were so severely impaired that attendance of compulsory Kindergarten was uncertain. Parents were deeply involved in accommodating their child’s rituals. Because of the children’s young age, medication was not indicated. Therefore, a minimal CBT intervention for parents was offered, mainly focusing on reducing family accommodation. Parents were asked to bring video tapes of critical situations that were watched together. They were coached to reduce family accommodation for OCD, while enhancing praise and reward for adequate behaviors of the child. CY-BOCS scores at the beginning and after 3 months show an impressive decline in OCD severity that remained stable after 6 months. At 3 months follow-up, all children were able to attend Kindergarten daily, and at 6 months follow-up, every child was admitted to the next level / class.

**Conclusions:**

Disseminating knowledge about the clinical presentation, diagnosis and treatment of early OCD may shorten the long delay between first OCD symptoms and disease-specific treatment that is reported as main predictor for persistent OCD.

## Background

Paediatric obsessive compulsive disorder [1] is a chronic condition with lifetime prevalence estimates ranging from 0.25 [2] to 2–3% [3]. OCD is often associated with severe disruptions of family functioning [4] and impairment of peer relationships as well as academic performance [5]. Mean age of onset of early onset OCD is 10.3 years, with a range from 7.5 to 12.5 years [6] or at an average of 11 years [7]. However, OCD can manifest itself also at a very early age - in a sample of 58 children, mean age of onset was 4.95 years [8], and in a study from Turkey, OCD is described in children as young as two and a half years [9]. According to different epidemiological surveys the prevalence of subclinical OC syndromes was estimated between 7 and 25%, and already very common at the age of 11 years [10].

Understanding the phenomenology of OCD in young children is important because both an earlier age of onset and a longer duration of illness have been associated with increased persistence of OCD [11–13]. One of the main predictors for persistent OCD is duration of illness at assessment, which underlines that early recognition and treatment of the disorder are crucial to prevent chronicity [10, 14, 15]. OCD in very young children can be so severe that it has to be treated in an inpatient-clinic [16]. This might be prevented if the disorder were diagnosed and treated earlier.

In order to disseminate knowledge about early childhood OCD, detailed descriptions of its phenomenology are necessary to enable clinicians to recognize and assess the disorder in time. Yet, studies on this young population are scarce and differ in the definition of what is described as ‘very young’. For example, 292 treatment seeking youth with OCD were divided into a younger group (3–9 years old) and an older group (10–18 years old) [17]. While overall OCD severity did not differ between groups, younger children exhibited poorer insight, increased incidence of hoarding compulsions, and higher rates of separation anxiety and social fears than older youth. It is not clear how many very young children (between 3 and 5 years old) were included in this study. Skriner et al. [18] investigated characteristics of 127 young children (from 5 to 8) enrolled in a pilot sample of the POTS Jr. Study. These young children revealed moderate to severe OCD symptoms, high levels of impairment and significant comorbidity, providing further evidence that symptom severity in young children with OCD is similar to that observed in older samples. To our knowledge, the only European studies describing OCD in very young children on a detailed, phenotypic level are a single-case study of a 4 year old girl [16] and a report from Turkey on 25 children under 6 years with OCD [9]. Subjects were fifteen boys and ten girls between 2 and 5 years old. Mean age of onset of OCD symptoms was 3 years, with some OCD symptoms appearing as early as 18 months of age. All subjects had at least one comorbid disorder; the most frequent comorbidity was an anxiety disorder, and boys exhibited more comorbid diagnoses than girls. In 68% of the subjects, at least one parent received a lifetime OCD diagnosis. The study reports no further information on follow-up or treatment of these young patients.

In comparison to other mental disorders, duration of untreated illness in obsessive compulsive disorder is one of the longest [19]. One reason may be that obsessive-compulsive symptoms in young children are mistaken as a normal developmental phase [20]. Parents as well as professionals not experienced with OCD may tend to ‘watch and wait’ instead of asking for referral to a specialist, thus contributing to the long delay between symptom onset and assessment / treatment [10]. This might ameliorate if health professionals become more familiar with the clinical presentation, diagnosis and treatment of the disorder in the very young. The purpose of this study is to provide a detailed description of the clinical presentation, diagnosis and treatment of OCD in five very young children.

## Case presentation

We describe a prospective 6 month follow-up of five cases of OCD in very young children (between 4 and 5 years old) who were referred to the OCD Outpatient Treatment Unit of a Psychiatric University Hospital. Three patients were directly referred by their parents, one by the paediatrician and one by another specialist. Parents and child were offered a first session within 1 week of referral. An experienced clinician (V.B.) globally assessed comorbidity, intelligence and functioning, and a CY-BOCS was administered with the parents.

### Instruments

To assess OCD severity in youth, the *Children Yale-Brown Obsessive Compulsive Scale CY-BOCS* [21] is regarded as the gold standard, with excellent inter-rater and test-retest reliability as well as construct validity [21, 22]. The CY-BOCS has been validated in very young children by obtaining information from the parent. As in the clinical interview Y-BOCS for adults, severity of obsessions and compulsions are assessed separately. If both obsessions and compulsions are reported, a score of 16 is regarded as the cut-off for clinically meaningful OCD. If only compulsions are reported, Lewin et al. [23] suggest a cut-off score of 8. In their CY-BOCS classification, a score between 5 and 13 corresponds to mild symptoms / little functional impairment or a Clinical Global Impression Severity (CGI-S) of 2. A score between 14 and 24 corresponds to moderate symptoms / functioning with effort or a CGI-S of 3. Generally, it is recommended to obtain information from both child and parents. However, in case of the very young patients presented here, CY-BOCS scores were exclusively obtained from the parents. The parents of all five children reported not being familiar with any obsessions their child might have. In accordance with previous recommendations [23], a cut-off point of 8 for clinically meaningful OCD was used.

### Patient vignettes

*Patient 1 is a 4 year old girl,* a single child living with both parents. She had never been separated an entire day from her mother. At the nursery, she suffered from separation anxiety for months. Parents reported that the girl had insisted on rituals already at the age of two. In the evening, she ‚had‘ to take her toys into bed and had got up several times crying because she ‚had to‘ pick up more toys. In the morning, only she ‚had the right‘ to open the apartment door. When dressing in the morning, she ‚had‘ to be ready before the parents. Only she was allowed to flush the toilet, even if it concerned toilet use of the parents. Moreover, only she ‘had the right’ to switch on the light, and this had to be with ten fingers at the same time. If she did not succeed, she got extremely upset and pressed the light button again and again until she was satisfied. The girl was not able to throw away garbage and kept packaging waste in a separate box. In the evening, she had to tidy her room for a long time until everything was ‚right‘. Whenever her routine was changed, she protested by crying, shouting and yelling at her parents. Moreover, she insisted on repeating routines if there had been a ‚mistake‘. In order to avoid conflict, both parents adapted their behavior to their daughter’s desires. In the first assessment with the parents, her score on the CY-BOCS was 15, implying clinically meaningful OCD. Psychiatric family history revealed that the mother had suffered from severe separation anxiety as a child and the father from severe night mares. Both parents described themselves as healthy adults.

*Patient 2 is a four and a half year old boy,* the younger of two brothers. He was reported to have been very oppositional since the age of two. Since the age of three, he insisted on a specific ritual when flushing the toilet – he had to pronounce several distinct sentences and then to run away quickly. Some months later he developed a complicated fare-well ritual and insisted on every family member using exactly the sentences he wanted to hear. If one of these words changed, he started to shout and threw himself on the floor. After a short time, he insisted on unknown people like the cashier at the supermarket to use the same words when saying good-bye.Moreover, he insisted that objects and meals had to be put back to the same place as before in case they had been moved. When walking outside, he had to count his steps and had to start this over and over again. In the morning, he determined where his mother had to stand and how her face had to look when saying good-bye. In order to avoid conflict, parents and brother had deeply accommodated their behavior to his whims. On the CY-BOCS, patient 2 reached a score of 15, which is equivalent to clinically meaningful OCD. Neither his father nor his mother reported any psychiatric disorder in past or present.

*Patient 3 is a 4 year old boy* referred because of possible OCD. Since the age of three, he had insisted on things going his way. When this was not the case, he threw a temper tantrum and demanded that time should be turned back. If, for example, he had cut a piece of bread from the loaf and was not satisfied with its form, he insisted that the piece should be ‘glued’ to the loaf again. Since he entered Kindergarten at the age of four, his behavior became more severe. If he was not satisfied with a certain routine like, for example, dressing in the morning, he demanded that the entire family had to undress and go to bed again, that objects had to lie at the same place as before or that the clock had to be turned back. In order to avoid conflict, the parents had repeatedly consented to his wishes. His behavior was judged as problematic at Kindergarten, because he demanded certain situations to be repeated or ‚played back‘. When the teacher refused to do that, the boy once run away furiously. On the CY-BOCS, patient 3 reached a score of 15. The mother described herself as being rather anxious (but not in treatment), the father himself as not suffering from any psychiatric symptoms. However, his mother had suffered from such severe OCD when he was a child that she had undergone inpatient treatment several times. This was also the reason why the parents had asked for referral to a specialist for the symptoms of their son.

*Patient 4 is a 5 year old girl,* the eldest of three siblings. Since the age of two, she was only able to wear certain clothes. For months, she refused to wear any shoes besides Espadrilles; she was unable to wear jeans and could only wear one certain pair of leggings. Wearing warm or thicker garments was extremely difficult, leading to numerous conflicts with her mother in winter. Socks had to have the same height, stockings had to be thin, and slips slack. When dressing in the morning, she regularly got angry and despaired and engaged in severe conflicts with her mother; dressing took a long time, whereas she had to be in Kindergarten on time. Her compulsions with clothes seemed to influence her social behavior as well; she had been watching other children at the playground for 40 min and did not participate because her winter coat did not ‚feel right‘. She started to join peers only when she was allowed to pull the coat off. She also had to dry herself excessively after peeing and was reported to be perfectionist in drawing, cleaning or tidying. Her CY-BOCS score was 15, equivalent to clinically meaningful OCD. Both parents described themselves as not suffering from any psychiatric problem in past or present. However, the grandmother on the mother’s side was reported to have had similar compulsions when she was a child.

*Patient 5 was a four and a half year old girl* referred because of early OCD. She had one elder brother and lived with both parents. At the age of 1 year, patient 5 was diagnosed with a benign brain tumor (astrocytoma). The tumor had been removed for 90% by surgery; the remaining tumor was treated with chemotherapy. The first chemotherapy at the age of 3 years was reasonably well tolerated. Shortly thereafter, the girl developed just-right-compulsions concerning her shoes. When the second chemotherapy (with a different drug) was started at the age of four, compulsions increased so dramatically that she was referred to our outpatient clinic by the treating oncologist. She insisted on her shoes being closed very tightly, her socks and underwear being put on according to a certain ritual, and her belt being closed so tightly that her father had to punch an additional hole. She refused to wear slack or new clothes and was not able to leave the toilet after peeing because ‘something might still come’; she used large amounts of toilet paper and complained that she wasn’t dry yet. She also insisted on straightening the blanket of her bed many times. She was described by her mother as extremely stressed, impatient and irritable; she woke up every night and insisted to go to the toilet, from where she would come back only after intense cleaning rituals. In the morning, she frequently threw a severe temper tantrum, including hitting and scratching the mother, staying naked in the bathroom and refusing to get dressed because clothes were not fitting ‚just right‘or were not tight enough. Shortly after the start of the second chemotherapy, the girl had entered Kindergarten which was in a different language than the family language. Moreover, her mother had just taken up a new job and had to make a trip of several days during the first month. Although the mother gave up her job after the dramatic increase in OCD severity, the girl’s symptoms did not change. As an association between chemotherapy and the increase in OCD symptoms could not be excluded, the treating oncologist decided to stop chemotherapy 2 weeks after patient 5 was presented with OCD at our department. At the moment of presentation, she arrived at Kindergarten too late daily, after long scenes of crying and shouting, or refused to go altogether. She reached a score of 20 on the CY-BOCS, the highest score of the five children presented here. Her father described himself as free of any psychiatric symptoms in past or present. Her mother had been extremely socially anxious as a child.

None of the siblings of the children described above was reported to show any psychiatric symptoms in past or present (Table 1).

**Table 1:** Five cases of early childhood OCD

Name	Age	Sex	CY-BOCS first session	CGI-S first session	Clinical presentation of compulsions	Comorbidities	Family History
Patient 1	4	f	15	3	Repetitive behaviour, symmetry, hoarding, tydying	Separation anxiety	Mother social anxious child
Patient 2	4	m	15	3	Counting steps, pronouncing certain phrases, fare-well ritual, repeating or winding back of situations	Lisping	Parents report no psychopathology
Patient 3	4	m	15	3	Repeating or winding back of situations, winding back time	Stuttering	Mother anxious, grandmother f.s. severe OCD
Patient 4	5	f	15	3	Just-Right-compulsions with clothings and shoes, excessive cleaning and drying rituals after urinating, tydying, symmetry	Separation anxiety	Parents report no psychopathology, grandmother m.s. light OCD
Patient 5	4	f	20	3	Just-Right-compulsions with clothings and shoes, excessive cleaning and drying rituals after urinating, tydying, symmetry	Separation anxiety, Astrocytoma	Mother social anxious child

*CGI-S* Clinical Global Impression Severity Scale, *CY-BOCS* Children’s Yale-Brown Obsessive Compulsive Scale, *f.s.* father’s side, *m.s.* mother’s side

The five cases described above show a broad range of OCD symptomatology in young children. Besides Just-Right compulsions concerning clothes, compulsive behavior on the toilet was reported such as having to pee frequently, having to dry oneself over and over again as well as rituals concerning flushing. Other symptoms were pronouncing certain words or phrases compulsively, insisting on a ‘perfect’ action and claiming that time or situations must be played back like a video or DVD if the action or situation were not ‘perfect enough’. The patients described here have in common that parents were already much involved in the process of family accommodation. For example, the parents of patient 3 had consented several times to undress and go to bed again in order to ‘play back’ certain situations; they had also consented turning back the clock in the house. The parents of patient 2 had accommodated his complicated fare-well ritual, thus having to rush to work in the morning themselves. However, all parents were smart enough not just to indulge their child’s behavior, but to seek professional advice.

### Treatment recommendations

Practice Parameters and guidelines for the assessment and treatment of OCD in older children and adolescents recommend cognitive behavior therapy (CBT) as first line treatment for mild to moderate cases, and medication in addition to CBT for moderate to severe OCD [24, 25]. However, there is a lack of treatment studies including young children with OCD [26]. A case series with seven children between the age of 3 and 8 years diagnosed with OCD describes an intervention adapted to this young age group. Treatment emphasized reducing family accommodation and anxiety-enhancing parenting behaviors while enhancing problem solving skills of the parents [27]. A much larger randomized clinical trial for 127 young children (5 to 8 years of age) with OCD showed family-based CBT superior to a relaxation protocol for this age group [14]. Despite these advances in treatment for early childhood OCD, availability of CBT for paediatric OCD in the community is scarce due to workforce limitations and regional limitations in paediatric OCD expertise [28]. This is certainly not only true for the US, but for most European countries as well.

When discussing treatment of OCD in young children, the topic of family accommodation is of utmost importance. Family accommodation, also referred to as a ‘hallmark of early childhood OCD’ [15] means that parents of children with OCD tend to accommodate and even participate in rituals of the affected child. In order to avoid temper tantrums and aggressive behavior of the child, parents often adapt daily routines by engaging in child rituals or facilitating OCD by allowing extra time, purchasing special products or adapting family rules and organisation to OCD [29–31]. Although driven by empathy for and compassion with the child, family accommodation is reported to be detrimental because it further reinforces OCD symptoms and avoidance behavior, thus enhancing stress and anxiety [4, 32].

### Parent-oriented CBT intervention

At the moment of first presentation, the five children were so severely impaired by their OCD that attendance of (compulsory) Kindergarten was uncertain. All parents reported being utterly worried and stressed by their child’s symptoms and the associated conflicts in the family. However, no single family wanted an in-patient treatment of their child, and because of the children’s young age, medication was not indicated. Some families lived far away from our clinic and / or had to take care of young siblings.

Therefore, a CBT-intervention was offered to the parents, mainly focusing on reducing family accommodation. This approach is in line with current treatment recommendations to aggressively target family accommodation in children with OCD [15]. Parents and child were seen together in a first session. The following sessions were done with the parents only, who were encouraged to bring video tapes of critical situations. The scenes were watched together and parents were coached to reduce family accommodation for OCD, while enhancing praise and reward for adequate behaviors of the child. Parents were also encouraged to use ignoring and time-out for problematic behaviors. As some families lived far away and had to take care of young siblings as well, telephone sessions were offered as an alternative whenever parents felt the need for it. Moreover, parents were prompted to facilitate developmental tasks of their child such as attending Kindergarten regularly, or building friendships with peers. The minimal number of treatment sessions was four and the maximal number ten, with a median of six sessions.

Three of the five children (patients 3, 4 and 5) were raised in a different language at home than the one spoken at Kindergarten. This can be interpreted as an additional stressor for the child, possibly enhancing OCD symptoms. Instead of expecting their child to learn the foreign language mainly by ‚trial and error‘, parents were encouraged to speak this language at home themselves, to praise their child for progress in language skills and to facilitate playdates with children native in the foreign language.

### Follow-up

Three and six months after intake, assessment of OCD-severity by means of the CY-BOCS was repeated. Table 2 shows an impressive decline in OCD-severity after 3 months that remained stable after 6 months. At 3 months follow-up, all children were able to attend Kindergarten daily, and at 6 months follow-up, every child was admitted to the next level of Kindergarten or, in the case of patient 4, to school.

**Table 2:** CGI-S and CY-BOCS scores at intake, 3 months and 6 months follow-up

Name	Age	Sex	CGI-S first session	CY-BOCS first session	CY-BOCS 3 months	CY-BOCS 6 months	CGI-S 6 months
Patient 1	4	f	3	15	0	0	0
Patient 2	4	m	3	15	5	7	1
Patient 3	4	m	3	15	9	7	1
Patient 4	5	f	3	15	x	4	1
Patient 5	4	f	3	20	13	4	1

*CGI-S* Clinical Global Impression Severity Scale, *CY-BOCS* Children’s Yale-Brown Obsessive Compulsive Scale

× Because of serious illness of the grandfather in the meantime, it was not possible to administer the CY-BOCS at 3 months follow-up. However, the interview at 6 months follow-up revealed that OCD symptom reduction had been progressive and comparable to that of the other children, also with regard to regular attendance of Kindergarten

## Discussion

We report on five children of 4 and 5 years with very early onset OCD who were presented at a University Department of Child and Adolescent Psychiatry. These children are ‚early starters‘with regard to OCD. As underlined in a recent consensus statement [10], delayed initiation of treatment is seen as an important aspect of the overall burden of OCD (see also [19]). In our small sample, a CBT-based parent-oriented intervention targeting mainly family accommodation led to a significant decline in CY-BOCS scores after 3 months that was maintained at 6 months. At 3 months, all children were able to attend Kindergarten daily, and at 6 months, every child was admitted to the next grade. This can be seen as an encouraging result, as it allowed the children to continue their developmental milestones without disruptions, like staying at home for a long period or following an inpatient treatment that would have demanded high expenses and probably led to separation problems at this young age. Moreover, the reduction on CY-BOCS scores was reached without medication. The number of sessions of the CBT-based intervention with the parents varied between four and ten sessions, depending on the need of the family. Families stayed in touch with the therapist during the 6 month period and knew they could get an appointment quickly when needed.

A possible objection to these results might be the question of differential diagnosis. Couldn’t the problematic behaviors described merely be classified as benign childhood rituals that would change automatically with time? As described in the patient vignettes, the five children were so severely impaired by their OCD that attendance of Kindergarten – a developmental milestone – was uncertain. Moreover, parents were extremely worried and stressed by their child’s symptoms and associated family conflicts. In our view, it would have been a professional mistake to judge these symptoms as benign rituals not worthy of diagnosis or disorder-specific treatment. One possible, but rare and debated cause of OCD are streptococcal infections, often referred to as PANS [33]. However, in none of the cases parents reported an abrupt and sudden onset of OCD symptoms after an infection. Instead, symptoms seem to have developed gradually over a period of several months or even years. In the case of patient 5 with the astrocytoma, first just-right compulsions appeared at the age of three (after the first chemotherapy), and were followed by more severe compulsions at the age of four, when – within a period of 6 weeks – a new chemotherapy was started, the mother took up a new job and the patient entered Kindergarten. Diagnosing the severe compulsions of patient 5 as, for example, adjustment disorder due to her medical condition would not have delivered a disorder-specific treatment encouraging parents to reduce their accommodation. This might have led to even more family accommodation and to more severe OCD symptoms in the young girl. Last but not least, a possible objection might be that the behaviors described were stereotypies. However, stereotypies are defined as repetitive or ritualistic movements, postures or utterances and are often associated with an autism spectrum disorder or intellectual disability. The careful intake with the children revealed no indication for any of these disorders.

Data reported here have several limitations. The children did not undergo intelligence testing; their reactions and behavior during the first session, as well as their acceptance and graduation at Kindergarten were assumed as sufficient to judge them as average intelligent. Comorbidities were assessed according to clinical impression and parents’ reports. The CBT treatment was based on our clinical expertise as a specialized OCD outpatient clinic. It included parent-oriented CBT elements, but did not have a fixed protocol and was adjusted individually to the needs of every family. Last but not least, no control group of young patients without an intervention was included.

## Conclusions and clinical implications

We described a prospective 6 month follow-up of five cases of OCD in very young children. At the moment of first presentation, all children were so severely impaired that attendance of Kindergarten was uncertain. Parents were deeply involved in accommodating their child’s rituals. Because of the children’s young age, medication was not indicated. Therefore, a minimal CBT intervention for parents was offered, mainly focusing on reducing family accommodation. CY-BOCS scores at the beginning and after 3 months show an impressive decline in OCD severity that remained stable after 6 months. At 3 months follow-up, all children were able to attend Kindergarten daily, and at 6 months follow-up, every child had been admitted to the next grade. OCD is known to be a chronic condition. Therefore, in spite of treatment success, relapse might occur. However, as our treatment approach mainly targeted family accommodation, parents will hopefully react with less accommodation, should a new episode of OCD occur. Moreover, parents stay in touch with the outpatient clinic and can call when needed.

The clinical implications of our findings are that clinicians should not hesitate to think of OCD in a young child when obsessive-compulsive symptoms are reported. The assessment of the disorder should include the CY-BOCS, which has been validated in very young children by obtaining information from the parent. If CY-BOCS scores are clinically meaningful (for young children, a score above 8), a parent-based treatment targeting family accommodation should be offered.

By disseminating knowledge about the clinical presentation, assessment and treatment of early childhood OCD, it should be possible to shorten the long delay between first symptoms of OCD and disease-specific treatment that is reported as main predictor for persistent OCD. Early recognition and treatment of OCD are crucial to prevent chronicity [14, 15]. As children and adolescents with OCD have a heightened risk for clinically significant psychiatric and psychosocial problems as adults, intervening early offers an important opportunity to prevent the development of long-standing problem behaviors [10, 19].

## Data Availability

All data generated or analyzed during this study are included in this published article [and its supplementary information files].

## Abbreviations

OCD: Obsessive compulsive behavior
CY-BOCS: Child Yale-Brown Obsessive Compulsive Scale
CBT: Cognitive Behavior Therapy
